# Do Male and Female Cowbirds See Their World Differently? Implications for Sex Differences in the Sensory System of an Avian Brood Parasite

**DOI:** 10.1371/journal.pone.0058985

**Published:** 2013-03-27

**Authors:** Esteban Fernández-Juricic, Agustin Ojeda, Marcella Deisher, Brianna Burry, Patrice Baumhardt, Amy Stark, Amanda G. Elmore, Amanda L. Ensminger

**Affiliations:** 1 Department of Biological Sciences, Purdue University, West Lafayette, Indiana, United States of America; 2 Department of Biological Sciences, California State University Long Beach, Long Beach, California, United States of America; Universität Bielefeld, Germany

## Abstract

**Background:**

Male and female avian brood parasites are subject to different selection pressures: males compete for mates but do not provide parental care or territories and only females locate hosts to lay eggs. This sex difference may affect brain architecture in some avian brood parasites, but relatively little is known about their sensory systems and behaviors used to obtain sensory information. Our goal was to study the visual resolution and visual information gathering behavior (i.e., scanning) of brown-headed cowbirds.

**Methodology/Principal Findings:**

We measured the density of single cone photoreceptors, associated with chromatic vision, and double cone photoreceptors, associated with motion detection and achromatic vision. We also measured head movement rates, as indicators of visual information gathering behavior, when exposed to an object. We found that females had significantly lower density of single and double cones than males around the fovea and in the periphery of the retina. Additionally, females had significantly higher head-movement rates than males.

**Conclusions:**

Overall, we suggest that female cowbirds have lower chromatic and achromatic visual resolution than males (without sex differences in visual contrast perception). Females might compensate for the lower visual resolution by gazing alternatively with both foveae in quicker succession than males, increasing their head movement rates. However, other physiological factors may have influenced the behavioral differences observed. Our results bring up relevant questions about the sensory basis of sex differences in behavior. One possibility is that female and male cowbirds differentially allocate costly sensory resources, as a recent study found that females actually have greater auditory resolution than males.

## Introduction

When animals communicate, the sender emits a signal that is then detected and processed by the receiver, which ultimately responds behaviorally. Much attention has been devoted to the sender’s and receiver’s behavioral interactions [1] and to some degree the neural architecture behind those interactions [2], [3]. However, we know relatively less about how the configuration of the receiver’s sensory system constrains the ability to detect and process signals [4] and how individuals allocate attention to different sensory components of a signal [5].

Differences in the sensory system of males and females have been reported in some vertebrate species [6], [7], [8]. However, little is known as to how these sex differences can influence behaviors associated with gathering sensory information. For instance, female *Sceloporus graciosus* lizards can detect the fast motion stimuli of male courtship signals more quickly than males [9], and they also spend more time orienting towards courtship displays with complex motion patterns [10]. Understanding sex differences in both the sensory system and information gathering behaviors is key to testing the mechanisms behind some signal evolution hypotheses (e.g., perceptual variability hypothesis [11]) as well as establishing the differential investment of males and females in different sensory modalities [12].

Our goal was to test for sex differences in visual resolution (i.e., cone photoreceptor density) and visual information gathering behaviors (i.e., head movements) in brown-headed cowbirds (*Molothrus ater*). Cowbirds are brood parasites, making them good models to study sex differences because (a) selection pressures vary between sexes (i.e., males’ role is limited to mate attraction and copulation without providing parental care or territories, whereas only females search for hosts to lay their eggs [13]), (b) males and females differ in their auditory systems (i.e., females have better auditory resolution [14]), and (c) males and females differ in their vigilance behavior while foraging in groups [15].

First, we studied the density of cone photoreceptors associated with chromatic (i.e., single cones [16], [17]) and achromatic vision/motion detection (double cones [18], [19], [20], [21]) in two parts of the retina: the center and the periphery. In brown-headed cowbirds, the fovea is approximately at the center of the retina and projects laterally [22], and parts of the retinal periphery (i.e., temporal region) project towards the binocular visual field [23]. Areas with higher cone photoreceptor density are expected to have higher visual resolution, and thus higher visual performance [24].

Second, we conducted a behavioral experiment exposing female and male cowbirds to an object with high and low chromatic saliency and measured how they gathered visual information with head movements. Visually-guided animals actively modify the position of their visual apparatus (i.e., eye, hence retina) to enhance the quality and quantity of the sensory information they can gather [25], [26], [27]. The avian fovea generally projects laterally in those species with central foveae because of the lateral position of the orbits in the skull. Consequently, when birds fixate on an object, they mostly move their heads (due to their comparatively limited eye movements) sideways around the object of interest to get images of it with the fovea of each eye [28], [29]. Head movement rates have been proposed as an indirect proxy for different visual tasks (e.g., visual search, visual fixation) in birds [15], [30].

We considered two opposing predictions regarding sex differences in visual resolution in cowbirds. First, we expected that females would have higher visual resolution because they are the ones involved in nest searching behavior [13]. Second, we expected that females would have lower visual resolution due to their higher auditory resolution [14], because of the compensatory plasticity hypothesis by which different sensory modalities may have different energy allocation [31], [32] given that processing sensory information is costly [33]. Regarding gathering visual information, we hypothesized that individuals with lower visual resolution may need to actively compensate for the lower quality of the information obtained by their retinas (e.g. [34]). We proposed two alternatives for this compensatory mechanism based on how birds may explore objects visually [29]. First, if low visual resolution requires an increase in the time a given retina is exposed to the object to gather the necessary amount of information, then the sex with the lower visual resolution would have lower head movement rates than the sex with higher resolution. Second, if low visual resolution requires an increase in number of exposures of a given retina to the object to obtain the necessary amount of information, we predicted that the sex with the lower visual resolution would have higher head movement rates than the sex with the higher resolution.

## Methods

### Ethics Statement

All animal procedures were approved by the IACUC’s at California State University Long Beach (220) and Purdue University (Protocol no. 09–018).

### General Procedures

We studied sex differences in the density of cone photoreceptors between August and December 2008, and in scanning behavior between September and December 2010. Cowbirds were captured using mist-nets and Australian traps under the States of California (California Department of Fish and Game) and Indiana (Indiana Department of Natural Resources) and Federal (Fish and Wildlife Service) permits. We housed brown-headed cowbirds in indoor enclosures (0.61 m×0.76 m×0.60 m) under a 14∶10 hour light:dark cycle, and provided them with food *ad libitum* except during the preceding periods of food deprivation for the behavioral experiment (see below). Water was always available. For the density of photoreceptor component, cowbirds were euthanized with CO2 following guidelines established by IACUC.

### Density of Cone Photoreceptors

We used 20 adult brown-headed cowbirds (10 females, 10 males) captured from populations in Southern California. Individuals were euthanized within 24–48 hours of capture to minimize the effects that artificial lighting may have on the absorbance of oil droplets [35]. We first recorded individual body mass. We then chose one eye (right or left) at random, removed it, and measured its eye axial length with a digital caliper. We removed the retina following the methods described in detail in [36]. In brief, the eye was hemisected and the eyecup placed in a phosphate buffered saline PBS solution (Sigma Life Science, P4417-100TAB). The retina was extracted using fine paint brushes (2/0 round Princeton Art and Brush Co. 4359 R) to detach it from the retinal pigmented epithelium. The orientation of the retina was recorded during this procedure by using as a reference point the pecten, which is a pigmented and vascular structure in the avian retina [37]. We then made radial cuts to the retina to flatten it. In those cases in which the retina was torn, we used the other eye’s retina only if <30 min elapsed since the death of the individual. The retina was mounted and coverslipped on a microscope slide with the photoreceptor layer up and with a drop of PBS. Our goal was to compare photoreceptor densities between the foveal and non-foveal (i.e., retinal periphery) areas of the retina. We obtained samples from the center of the retina as this is where the fovea is localized in this species [22]. We decided to get samples from the retinal periphery in four different regions to avoid any bias: dorsal, ventral, temporal, and nasal. Therefore, we sampled from five 2.32 mm^2^ sampling regions. We pooled the data of the dorsal, ventral, temporal, nasal regions into a retinal periphery area.

The slide was placed in an Olympus BX51 microscope fitted with epifluorescent light (Olympus U-RFL-T), and a long-pass filter for viewing wavelengths longer than 420 nm. Samples were examined at a 40× magnification. We took pictures of each sampling area with a Moticam 2300 3.0 M pixel camera using Images Plus software Version 2.0 ML. Each of the five sampling regions (2.32 mm^2^) was divided into a grid of 8×8 frames (each frame was 0.036 mm^2^), yielding a total of 64 frames per sampling region. We then took pictures of 32 frames by starting from the upper left corner moving horizontally (and vertically at the end of each row of 8 frames) and skipping every other frame. In each frame, we took two pictures: one under the bright field and one under the epifluorescent field.

We distinguished the different types of photoreceptors using oil droplets, which are organelles in the avian retina that enhance color discrimination [38]. In birds, each cone photoreceptor is associated with a specific type of oil droplet. Single cone photoreceptors with ultraviolet-(UVS) visual pigments have transparent (T-type) oil droplets that do not absorb light in the visible range [39]. Single cone photoreceptors with a short wavelength-sensitive (SWS) visual pigment have colorless (C-type) oil droplets with cut-off wavelengths from 392 to 449 nm [39]. Single cone photoreceptors with a medium wavelength-sensitive (MWS) visual pigment have yellow (Y-type) oil droplets with cut-off wavelengths from 490 to 516 nm [39]. Finally, single cone photoreceptors with a long wavelength-sensitive (LWS) visual pigment have red (R-type) oil droplets with cut-off wavelength from 514 to 586 nm [39]. The double cone photoreceptors have a LWS visual pigment, with the principal member having a P-type oil droplet (cut-off wavelength varying from 407 to 419 nm [39]). We did not identify rod photoreceptors as it required the use of other methodological procedures [40].

We estimated the density of single and double cone photoreceptors by counting the number of oil droplets/mm^2^ at the center and periphery of the retina. We followed Hart’s [41] criteria to distinguish the different oil droplets (Appendix S1). Three observers counted the retina after extensive training led to differences of <5% among them. We did not correct for tissue shrinkage because we used fresh retinas. Some of the retina pictures obtained did not have any oil droplets on the whole picture or on specific parts of it. This could be an indication of loss of photoreceptors due to the use of a brush during the preparation (i.e., removal of the pigmented epithelium). Because of the potential bias this could generate in the calculation of the overall densities, we removed those pictures from the analysis.

### Head Movement Behavior

We used 20 adult brown-headed cowbirds (10 females, 10 males) captured from populations in Tippecanoe County, Indiana. We conducted the experiment indoors with fluorescent bulbs, which had a flicker rate of 20 kHz to minimize the potential confounding effects of artificial lighting [42]. A 0.5×0.5×0.4 m enclosure was on top of a 1-m high table (2 m under the lighting fixture). The enclosure was made of mesh wire with two sides made of Plexiglas to facilitate the recording of the focal’s behavior. The enclosure was sitting on a wooded bottom that was covered with light brown paper lining, which was replaced after each trial. At the center of the cage, we positioned a cube (4.5 cm side length) that was either colored (painted following the coloring pattern similar to a Rubik’s cube) or black. The top of the cube had a small hole where we placed about 1 g of millet seeds to grab the visual attention of the animal during the first few minutes of the trial. Food was not available in any other area of the cage. The experimental arena was surrounded by black cloth to minimize visual distractions while the animal was in the enclosure. We used three camcorders on tripods to record the focal from the sides and one camcorder was positioned on top of the enclosure to obtain a top-view of the animal.

Birds were food-deprived the night before (an average of 12.01±0.14 hrs) to enhance their motivation during the trials. The experiment was conducted at averaged light levels of 670.60±15.09 lux and temperatures of 22.10±0.10°C. None of these three factors significantly influenced head movement rates towards the cube (light levels, F_1,17_ = 0.06, P = 0.811; temperature, F_1,17_ = 2.21, P = 0.155; food deprivation, F_1,17_ = 0.79, P = 0.386); so they were excluded from the final models.

A trial consisted of a single cowbird exposed to a single cube (either colored or black) for 10 min. After the trial, animals were returned to the housing enclosures and food and water were provided *ad libitum*. Each individual was exposed to both conditions (colored and black cubes) in a random order.

We measured head movement rates of male and female cowbirds with JWatcher [43]. We analyzed the first 90 s of the videos, as it was the time cowbirds appeared more motivated to face the cube. We divided head movement behavior into bouts in which cowbirds’ eyes were towards the cube or away from the cube based on information on the configuration of its visual field [23] and our top-view camera. Head movement behavior towards the cube was defined as when the cowbird bill was within 120° in either the right or left direction of the cube; whereas head movement rate away from the cube was any other bill orientation. We did not distinguish between head movements with different amplitude because the degree of simultaneous calibration of the different cameras was not enough to obtain accurate measurements between trails. We also measured the proportion of time animals spent with their heads towards the cube (per definition above) as a potential confounding factor. We only used head movement rate towards the cube in our statistical analyses (see below).

### Perceptual Modeling

One of the factors that could be affecting the perception of the cubes by females and males is how salient they are from the visual background. Visual contrast models take into consideration (among other things) the reflectance of the background and the object, the spectral properties of the light, the sensitivity of the visual system (absorbance of visual pigments and oil droplets) and the *relative* density of photoreceptors [44]. Consequently, we estimated chromatic contrast of the colored and black cubes from the perspective of each individual male and female by calculating the *relative* densities of photoreceptors at the center and periphery of the retina (in relation to the UVS cone densities) based on the raw densities of photoreceptors measured for this study (see *Density of cone photoreceptors* section above). We obtained information on the absorbance of the visual pigments and oil droplets of brown-headed cowbirds (but without distinguishing between sexes), which have a UVS visual system, using microspectrophotometry (Appendix S2). We also measured the reflectance of the cubes, the irradiance of the fluorescent bulbs at the enclosure level, and the reflectance of the lining at the bottom of the enclosure (Appendix S3). This information allowed us to parameterize the chromatic contrast model for this particular species (details of calculations in Appendix S3). The only parameter that varied between sexes in our model was the *relative* density of photoreceptors. We did not calculate achromatic contrast because the model we used did not include double cone photoreceptor density as a parameter.

### Statistical Analysis

We first assessed sex differences in body mass and eye size with general linear models. We ran general linear mixed models to test for sex differences in the density of single cones (UVS, SWS, MWS, and LWS pooled together) and double cones. Besides sex, we also included retinal sector (central, periphery) and the interaction between sex and retinal sector as independent factors. Although our study was not designed to test for differences in cone density between the right and left eyes [45], we included eye and the interaction between eye and sex to control for this potential confounding factor. When an interaction effect was significant, we ran pairwise comparisons with t-tests. We used general linear mixed models to assess sex differences in head movement rate towards the cube, including cube type, the interaction between cube type and sex, and the proportion of time with the head towards the cube as independent factors. We analyzed differences in chromatic contrasts (measured in units of just noticeable differences or JND, Appendix S3) between sexes, cube types, and retinal sectors with general linear mixed models. We also included in this model the interactions between sex and cube type, and sex and retinal sector. In all general linear mixed models, we included focal ID in the model as a repeated measures factor. We checked for the normality of the residuals and homogeneity of variances in all models. Body mass and eye size analyses were conducted in Statistica 10; all other statistical analyses, in SAS 9.2.

## Results

### Body Mass and Eye Size

Previous studies have shown that brown-headed cowbird males are larger than females [15]. We confirmed this trend with the individuals used in the retinal analysis [females = 31.63±0.85 g, males = 39.05±1.06 g, F_1,18_ = 29.92, P<0.001] as well as the behavioral experiment [females = 36.36±1.49 g, males = 41.06±1.58 g, F_1,18_ = 4.68, P = 0.044]. However, we did not find significant differences in eye size measured as eye axial length [females = 6.58 mm, males = 6.66 mm, F_1,18_ = 0.55, P = 0.467].

### Density of Photoreceptors

**Figure 1:**
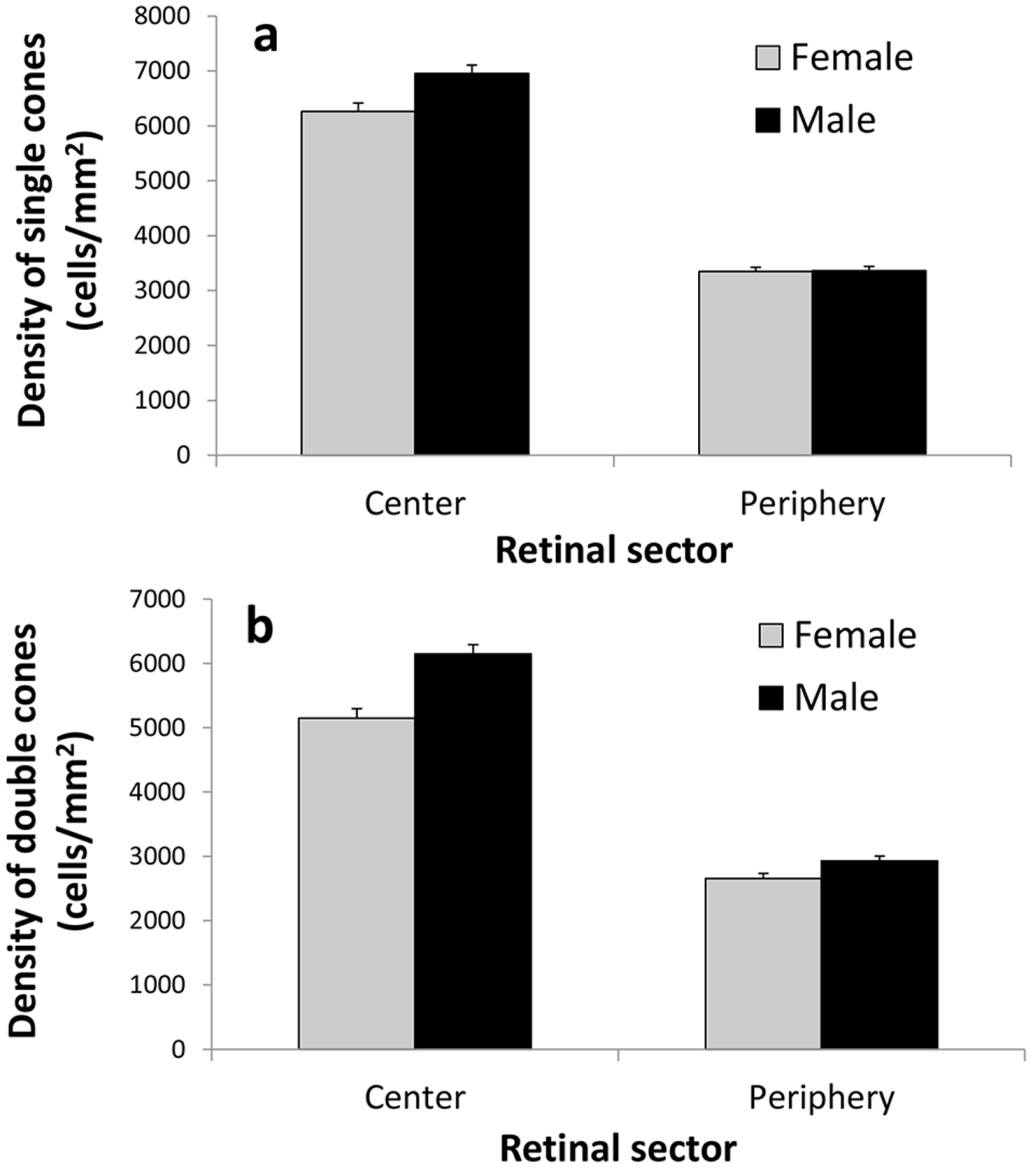
Variations in the density of brown-headed cowbird female and male photoreceptors. Panel (a) shows the density of single cones, and panel (b) shows the density of double cones at the center and periphery of the retina.

The density of single cones varied significantly between sexes and retinal sectors (Table 1). Overall, males (5,160.75±84.37 cells/mm^2^) had a significantly higher density of single cones than females (4,804.25±88.66 cells/mm^2^). Additionally, the density of single cones was higher at the center (6,609.46±106.97 cells/mm^2^) than at the periphery (3,355.54±55.18 cells/mm^2^) of the retina. However, the difference between the sexes varied with the retinal sector, yielding a significant interaction effect (Table 1; Fig. 1a). Males had significantly higher single cone densities than females at the center of the retina (t_18_ = 3.12, P = 0.006; Fig. 1a), but this difference was not significant at the retinal periphery (t_18_ = 0.22, P = 0.830; Fig. 1a). We did not find significant effects of eye and the interaction between eye and sex on the density of single cones (Table 1).

**Table 1:** Results from general linear mixed models assessing the effects of sex, retinal sector, and other factors on the density of single cones, double cones, and chromatic contrast.

	F	d.f.	*P*
*Single cone density*			
Sex	8.48	1, 16	**0.010**
Retinal sector	756.52	1, 18	**<0.001**
Eye	1.06	1, 16	0.319
Sex X Retinal sector	8.16	1, 18	**0.011**
Sex X Eye	1.52	1, 16	0.235
*Double cone density*			
Sex	29.73	1, 16	**<0.001**
Retinal sector	642.62	1, 18	**<0.001**
Eye	0.46	1, 16	0.579
Sex X Retinal sector	10.33	1, 18	**0.005**
Sex X Eye	1.32	1, 16	0.267
*Chromatic contrast*			
Sex	0.91	1, 18	0.354
Cube	472.56	1, 18	**<0.001**
Retinal sector	21.85	1, 18	**<0.001**
Sex X Cube	0.38	1, 18	0.545
Sex X Retinal sector	0.01	1, 18	0.905

Significant results are marked in bold.

The density of double cones also varied between sexes and retinal sectors (Table 1), with males (4,538.33±80.33 cells/mm^2^) having significantly higher density than females (3,902.98±84.41 cells/mm^2^), and the retinal center (5,648.31±101.85 cells/mm^2^) having significantly higher density than the retinal periphery (2,793.00±52.54 cells/mm^2^) of the retina. Yet, the differences between sexes varied significantly with retinal sector giving rise to an interaction effect (Table 1; Fig. 1b). The difference in double cone density between males and females was more pronounced at the center (t_18_ = 4.75, P<0.001; Fig. 1b) than at the periphery (t_18_ = 2.60, P = 0.018; Fig. 1b) of the retina. We did not find significant effects of eye and the interaction between eye and sex on the density of double cones (Table 1).

We also investigated whether the sex differences in the *relative* density of cone photoreceptors (in relation to the UVS cones) could affect the ability of cowbirds to perceive the cubes they were exposed to in the behavioral experiment. We estimated the chromatic contrast of the two cubes for males and females at the center and periphery of the retina. Considering both retinal sectors, we did not find any significant difference between males (42.53±1.78 JNDs) and females (44.92±1.78 JNDs) in chromatic contrast (Table 1). Additionally, the interactions between sex and cube type, and sex and retinal sector were not significant (Table 1). We found that cowbirds would perceive the colored (71.02±1.78 JNDs) cube as more chromatically contrasting than the black (16.43±1.78 JNDs) cube (Table 1). Furthermore, cowbirds would perceive either cube as significantly more contrasting with the center (49.60±1.78 JNDs) than with the periphery (37.86±1.78 JNDs) of their retinas (Table 1).

### Head Movement Behavior

**Figure 2:**
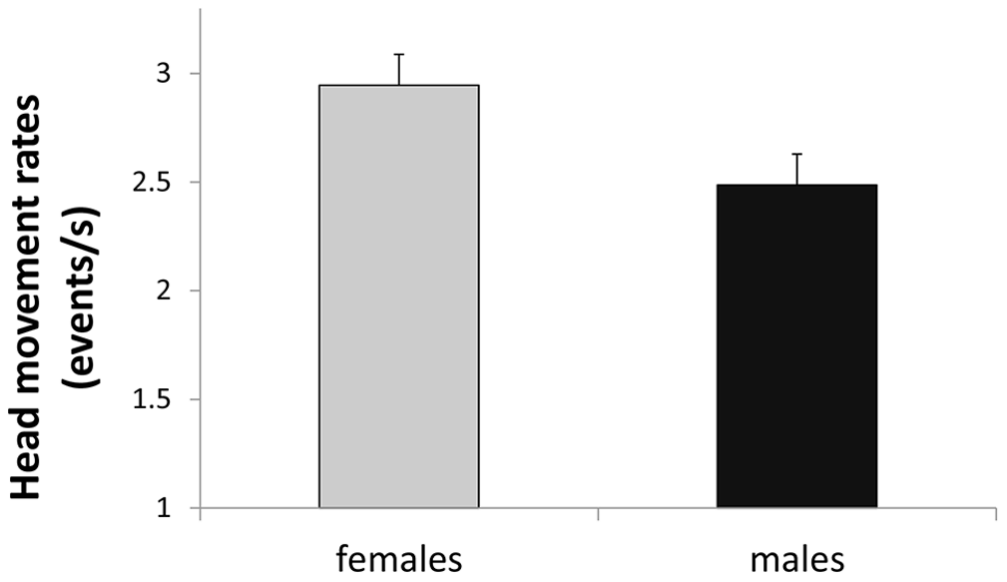
Variation in the scanning behavior (head movement rate) between female and male brown-headed cowbirds.

Head movement behavior differed between sexes. Females showed significantly higher head movement rates towards the cube than males (F_1, 18_ = 5.16, P = 0.036; Fig. 2), accounting for the proportion of time with the head oriented towards the cube (F_1, 17_ = 7.66, P = 0.013). Neither the color of the cube (F_1, 17_ = 1.96, P = 0.179) nor the interaction between sex and cube color yielded significant effects (F_1, 17_ = 1.53, P = 0.232).

## Discussion

We found that brown-headed cowbird females had lower density of cone photoreceptors associated with chromatic and achromatic/motion vision and higher head movement rates when gathering visual information than males. Although the cone photoreceptor data came from populations in Southern California, whereas the head movement behavior data came from populations in Indiana, similar sex differences in head movement rates were reported in a previous study [15]. Specifically, the time between consecutive head movements (i.e., the inverse of head movement rate) was shorter in cowbird females than males in an outdoors experiment varying the density of conspecifics [15]. The cowbirds used in Fernández-Juricic *et al.* [15] were also obtained from the same Southern California populations as the ones used for the cone photoreceptor data in the present study. Thus, we believe that our results may be representative of sex differences in visual resolution and visual information gathering behavior in this species. However, we cautioned that the potential link between physiology and behavior proposed below should be considered preliminary until future studies measuring both parameters on the same individuals are conducted. Nevertheless, our study does add new evidence on brown-headed cowbird sex differences besides the ones found in other components of its nervous system [46], [47], [14], as well as in other cowbird species (e.g., [48], [49]).

Visual resolution is affected by two main factors: eye size and the packing of photoreceptors and retinal ganglion cells [50]. We cannot rule out sex differences in ganglion cell density, but we did not find significant differences in eye size between females and males. At the photoreceptor level, we found that the overall distribution of cones matched the previously described distribution of retinal ganglion cells [22], with higher density of both single and double cones in the central retinal region around the fovea. Thus, the fovea can be considered the center of both chromatic and achromatic/motion vision in brown-headed cowbirds.

Cowbird females had 12.5% lower density of both cones types in the foveal area, and 10.3% lower density of double cones in the retinal periphery, compared to males. Future studies should determine if these photoreceptor differences also translate into differences at the retinal ganglion cell layer, which is responsible for the transfer of information from the retina into the visual centers of the brain [51]. Following Pettigrew *et al.* [52] and Williams & Coletta [53] and pooling the densities of both cone types, these sex differences would translate into a 7.6% reduction in the foveal visual acuity of females compared to that of males. Females are also expected to have lower performance in achromatic vision and motion tasks than males. Previous studies on the human visual system found sex differences in the relative ratios of LWS and MWS photoreceptors [54], which could affect the thresholds of color discrimination [55]. Additionally, males and females of some cichlid species differ in cone opsin gene expression and frequency of cone pigments, which could influence the ability to discriminate between potential mates [56].

The difference in cone density between the center and the periphery of the retina appears to be an important factor driving the degree of eye and head movements to sample different parts of the visual space with the high visual resolution of the fovea [57], [30]. Cowbird female head movement rates were 15.6% higher than those of males when exposed to an object in a visually controlled environment. These behavioral differences may be related to the way females perceive predation risk in relation to males; however, the study was conducted indoors where the expectation of a predator attack may be considered lower. Another possibility is that these differences at the behavioral level may be associated with our findings at the photoreceptor level. If so, female cowbirds may compensate for their lower visual resolution (i.e., lower density of single and double cones) with a different visual exploratory strategy than males. By exposing the right and left foveae to an object alternatively in quicker succession, females may boost the quality of information obtained. This behavioral result supports the view that visual fixation in birds encompasses gazing alternatively with both foveae in quick succession [29] as opposed to species with frontally placed eyes that generally lock their gaze on objects for a relatively longer period of time [58]. Similar sex differences were found in red back salamanders (*Plethodon cinereus*), where females nose-tap more frequently probably due to their smaller vomeronasal organs compared to that of males [59], [60]. This suggests that the sex with the sensory modality with lower resolution requires more sensory information sampling.

Another alternative is that female cowbirds may have sampled more frequently because of differences in visual contrast between the object and the background rather than visual resolution per se. Yet, our estimates of chromatic contrast, based on the *relative* densities of single cone photoreceptors (rather than the raw cone densities described above), did not yield any significant sex effects using for the first time a model parameterized for the brown-headed cowbird visual system. Our chromatic contrast model did not include sex-specific estimates of the sensitivity of oil droplets due to sample size constraints, which could have influenced chromatic contrast estimates. We studied only one visual dimension (e.g., density of single and double cones) that could be associated with the sex differences in visual exploratory behavior; however, there are other dimensions that deserve further study. For instance, sexes may also differ in the arrangement of cone photoreceptors [61], temporal visual resolution [62], ganglion cell density [63], and the wiring between the photoreceptor and ganglion cell layers that can influence motion detection [51].

The reason behind female cowbirds having lower density of photoreceptors than males cannot be answered with our data. Many factors may be involved, such as differential adult predation, use of different cues during mate choice, host searching behavior, etc. The sex differences in the visual system and visual information gathering behavior of cowbirds could be also considered in the context of a previous study that found that female cowbirds have better auditory ability to discriminate frequency and pure tones than males [14]. We speculate that females may reduce the costs of sensory processing by investing more sensory resources in the auditory system than in the visual system, probably to enhance the likelihood of finding suitable hosts during nest searching [64], in which males are not involved. Females obtain information on male quality from mostly the vocal contents of their audio-visual displays [65], [66], although the presence of the visual component (i.e., wing-spread) also stimulates them sexually [67], [68]. Females would not be challenged to resolve visually this wing-spread component because males generally display at very close distances (<0.5 m) [69]. Interestingly, female egg laying in some related cowbird species occurs before sunrise [70], when cone photoreceptors may not be necessarily activated due to low light levels.

Males on the other hand also give male-directed audio-visual displays whose visual components (e.g., depth of the bowing motion) are more intense than those in the female-directed version [71]. Males use these displays to establish dominance hierarchies by assessing the rivals’ display rate and intensity [72]. One possibility is that cowbird males may benefit from having enhanced chromatic, achromatic, and motion vision abilities to quickly assess the fighting ability of competitors and avoid risky physical interactions [68]. Additionally, the higher visual capabilities of males may allow them to resolve more easily the fast and subtle wing strokes that females give at certain points during the male displays as a precursor of the copulatory posture [73]. This interpretation assumes that investment in sensory processing is costly [33], as found in other species that invest differentially across sensory modalities (e.g., [74]).

Our results bring up interesting questions about the sensory basis of differences in the behavior of males and females. If there are sex differences in brown-headed cowbird sensory physiology, as found in this and a previous study [14], it is likely that the sensory space of males and females varies, which would affect the quality and quantity of information available for higher order processing in the brain and ultimately behavior. Future work on species where sexes are under different selection pressures should explicitly test the degree to which different behaviors can compensate for lower sensory resolution and whether trade-offs between sensory modalities are the result of optimizing gathering information of different fitness value.

## Supporting Information

Appendix S1 **Criteria used to determine the different types of oil droplets.**
(DOC)

Appendix S2 **Sensitivity of the visual pigments and oil droplets of the brown-headed cowbird retina.**
(DOCX)

Appendix S3 **Perceptual modeling of the visual stimuli.**
(DOCX)
