# Supplementary material for: Do Male and Female Cowbirds See Their World Differently? Implications for Sex Differences in the Sensory System of an Avian Brood Parasite
Source: PLoS One. 2013 Mar 27;8(3):e58985. doi: 10.1371/journal.pone.0058985 (PMC3609808; doi:10.1371/journal.pone.0058985)
Supplement: Appendix S1 — Criteria used to determine the different types of oil droplets. (DOC) [file pone.0058985.s001.doc]

**Appendix S1: Criteria used to determine the different types of oil droplets**

We used oil droplets to differentiate among the different types of cone photoreceptors in the brown-headed cowbird retina. Oil droplets contain high lipid, light absorbing pigments, such as carotenoids (Hart and Hunt, 2007; Johnston and Hudson, 1976) that are present in the inner segment of the photoreceptor. Oil droplets act as light filters narrowing the spectral sensitivity of the cone photoreceptors (Goldsmith et al. 1984; Wortel and Nuboer, 1986).

We distinguished between five types of oil droplets, which are associated with different types of cone photoreceptors: T type (associated with the UVS single cone), C type (associated with the SWS single cone), Y type (associated with the MWS single cone), R type (associated with the LWS single cone), and P type (associated with the double cone). We used the relative size of the oil droplets and their coloration color under bright field and epifluorescent light to identify each type (Goldsmith et al. 1984; Hart et al.1998). In the next paragraphs and Table 1 we summarize the criteria used, which closely follows Hart (2001a, b).

The T-type (or transparent) oil droplet is the smallest oil droplet (1-2 microns; Hart, 2001b). Under bright field illumination T-type oil droplets have a dull greenish appearance, but they do not appear at all under epifluorescent light. The C-type (or colorless) oil droplets are the second smallest (2-3 microns). Under bright field illumination, C-type oil droplets appear from pale yellow to sometimes having no color and showing only their circumference. Under the epifluorescent light, C type oil droplets appear bright whitish-yellow or bluish-green. Due to their relative size, it was more effective to use the epifluorescent light pictures to distinguish C-type oil droplets. Because T-type oil droplets do not appear under epifluorescent light, C-type oil droplets are the smallest under epifluorescent light compared to the other types.

The Y-type (or yellow) oil droplets measure approximately 3-3.5 microns (Hart, 2001b), which is approximately medium in size compared to the other types. The Y-type oil droplets appear under bright field illumination as dark golden yellow making them relatively easy to tell apart from the other types. Under epifluorescent light, Y-type oil droplets appear as dull, dark yellow in comparison to the dark background of the picture. We generally used the epifluorescent pictures to identify Y-type oil droplets due to their coloration. R-type (or red) oil droplets have approximately the same size as the Y-type oil droplets (3-3.5 microns; Hart, 2001b), but they look different under the microscope. Under bright field illumination, R-type oil droplets appear from bright red to a dark burgundy (brownish-red). Under epifluorescent light, R type oil droplets appear dark red (reddish-black). R-type oil droplets exposed to UV light for over a minute degrade quickly and the dark red color under epifluorescent light would change to a lighter redish-yellow color; therefore, we often used the bright field illumination to tell them apart.

P-type (or principal) oil droplets are visually the largest oil droplets (3.5-4.5 microns; Hart, 2001b). The appearance of P-type oil droplets under bright field illumination is dark yellowish or dark greenish. Under epifluorescent light the appearance of the P-type oil droplets varies from dull yellowish to greenish-yellow but sometimes they might appear bright tan yellowish. When categorizing P type oil droplets it is more advantageous to use the epifluorescent light, while taking into consideration both color and size of the oil droplet. We primarily used the size of the oil droplet, since they are the largest ones, and used the color to double-check our identification.

**Literature cited**

Goldsmith, T. H., Collins, J. S. & Licht, S. 1984. The cone oil droplets of avian retinas. Vision Research 24: 1661–1671.

Hart, N.S. 2001a. The visual ecology of avian photoreceptors. Progress in Retinal and Eye Research 20: 675-703.

Hart, N.S. 2001b. Variations in cone photoreceptor abundance and the visual ecology of birds. Journal of Comparative Physiology A 187: 685-697.

Hart, N. S. & Hunt, D. M. 2007. Avian visual pigments: characteristics, spectral tuning and evolution. The American Naturalist 169(1): S7-26.

Hart, N.S., Partridge, J.C. & Cuthill, I.C. 1998. Visual pigments, oil droplets and cone photoreceptor distribution in the European starling (*Strunus vulgaris*). Journal of Experimental Biology 201: 1433-1446.

Johnston, D. & Hudson, R. A. 1976. Isolation and composition ofthe carotenoid-containing oil droplets from cone photoreceptors. Acta Biochimica Biophys 424: 235–245.

Wortel, J. F. & Nuboer, J. F. 1986. The spectral sensitivity of blue sensitive pigeon cones: evidence for complete screening of the visual pigment by the oil-droplets. Vision Research 26: 885-886.

Table 1. Selection criteria for determining oil droplet types (based on Hart 2001). The relative size scale varies from 1 (smallest) to 4 (largest).

| **Oil droplet**  **type** | **Relative size** | **Bright field appearance** | **Epifluorescent light appearance** | **Bright field appearance (example)** | **Epifluorescent light appearance (example)** |
| --- | --- | --- | --- | --- | --- |
| T type (transparent) | 1 | Dull greenish | No appearance | 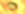 | 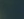 |
| C type  Colorless | 2 | Pale yellow to colorless | Bright whitish yellow to green bluish | 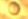 | 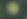 |
| Y type  (yellow) | 3 | Dark golden yellow | Dull dark yellow | 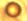 | 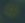 |
| R type  (red) | 3 | Bright red to a dark burgundy (browning red) | Dark red to reddish black | 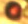 | 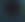 |
| P type  (principal) | 4 | Dark yellowish to dark greenish | Dull yellowish to greenish yellow and/or bright tan yellowish | 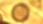 | 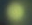 |
