# Supplementary material for: Do Male and Female Cowbirds See Their World Differently? Implications for Sex Differences in the Sensory System of an Avian Brood Parasite
Source: PLoS One. 2013 Mar 27;8(3):e58985. doi: 10.1371/journal.pone.0058985 (PMC3609808; doi:10.1371/journal.pone.0058985)
Supplement: Appendix S2 — Sensitivity of the visual pigments and oil droplets of the brown-headed cowbird retina. (DOCX) [file pone.0058985.s002.docx]

**Appendix S2: Sensitivity of the visual pigments and oil droplets of the brown-headed cowbird retina**

**Background**

Birds have two photosensitive components in the retina: visual pigments and oil droplets, in the outer and inner segments of the photoreceptors (Cuthill, 2006). The patterns of light absorption of the different types of visual pigments and oil droplets determines the sensitivity of the visual system of a species, which is relevant to estimate its color space. Microspectrophotometry is used to measure the sensitivity of visual pigments and oil droplets in the retina (Liebman, 1972). This is done by isolating photoreceptor outer segments and oil droplets and measuring the absorbance of these individual cellular components (Crescitelli, 1972). We conducted a microspectrophotometric analysis of the brown-head cowbird retina to characterize the visual space of this species. This analysis was not designed with the intention of establishing sex differences in the sensitivity of visual pigments and oil droplets, so we pooled the information from both sexes.

**Methods**

We did microspectrophotometry on six brown-headed cowbirds (4 females, 1 male, and 1 individual of unknown sex). One individual was processed in April, 2010 and five in April-May, 2012, within 4 to 12 days from capture in Tippecanoe County, IN, USA. We dark-adapted birds for a period of 1-3 hours to promote the development of higher concentrations of visual pigment in the cone outer segments. We then euthanized the bird with CO_2_ and processed one eye. We stored the additional eye on ice for 3-4 hours in a light shielded vial containing phosphate buffered saline (PBS; pH 7.2-7.4) and sucrose water. We randomized which eye (right, left) was processed first.

The retina extraction followed the procedures presented in Ullmann et al. (2012). We hemisected the eye using a razorblade, removed the vitreous humor from the eye cup, and separated the retina carefully from the pigmented epithelium and sclera using a small paintbrush. We removed an approximately 6 mm^2^ piece of retina, placed it onto a Corning No. 1 22 x 30 mm glass slide, and macerated it with a razor blade. We then covered the macerated retina with a Corning No. 1 18 mm^2^ cover slip, sealing it to the glass slide using black nail lacquer to prevent desiccation. All tissue processing and microspectrophotometric measurements were performed in either infrared or dim red light to prevent the bleaching of the visual pigments.

We took measurements on a custom-made microspectrophotometer (Dr. Ellis Loew, Cornell University, Ithaca, NY; designed described in McFarland and Loew, 1994). We measured the absorbance (i.e., amount of light absorbed as light passes through a substance; Liebman, 1972) of the cone and rod outer segments as well as the inner segment oil droplets. We used a Zeiss Ultrafluar Glyc objective (32x, NA 0.4) as the condenser and a dry objective (80x, NA 0.9). We viewed the cellular components on an EXVision Super Circuits CCD camera attached to an 8” TFT Color LCD Monitor that we covered in red Plexiglas. After identifying an isolated outer segment or oil droplet, we took a baseline measurement in an empty area of the preparation. We then took a measurement of the absorbance of the visual pigment or oil droplet in 1nm increments from 350-750 nm in the individual from 2010 and from 375-750 nm in the five individuals from 2012. We changed the wavelength range due to a large absorbance spike originating from the light source.

When we measured cone or rod outer segments, we tried to bleach the pigment for 60 s to confirm its identity. Bleaching is essential as it allows us to distinguish the visual pigment, which degrades in white light, from photo-reactive substances in the preparation that do not degrade in light (Liebman, 1972). For the analysis, we only used outer segment spectra that we were able to bleach. To differentiate the MWS from the RH1 (rod) spectra, which are nearly identical, we classified these spectra based on the morphological characteristics of the outer segments we measured. Rod outer segments are large, rectangular shaped, and contain conspicuous horizontal striations, while MWS outer segments are small, triangular shaped, with no conspicuous striations (Crescitelli, 1972). Oil droplets do not bleach when exposed to white light, so we distinguished them from other cellular components by their shape.

We determined the wavelength-specific peak sensitivity of the outer segment visual pigments (λ_max_) by fitting A1-rhodopsin pigment templates to the normalized absorbance spectra of each visual pigment using the MSP Control and Analysis Program (Ellis Loew & Mary Stauble © 1988-92). Visual pigment spectra can be completely characterized by a single parameter λ_max_, as their shape is highly conserved across pigment types and animal taxa (Govardovskii et al. 2000). Oil droplets, however, require the use of several parameters to describe the shape of their absorbance curves as they are highly dependent on the concentration and composition of carotenoids (Hart, 2001a). The most commonly used parameters to characterize oil droplets are: λ_cut_, the wavelength at which 100% of the light is absorbed by the oil droplet; λ_mid_, the wavelength at which 50% of the light is absorbed, and λ_0_, the wavelength at which 63% of light is absorbed (classically the wavelength at which the transmittance equals 1/e) (Lipetz, 1984; Hart and Vorobyev, 2005). We determined these parameters using an oil droplet spectra analysis program written in MATLAB (The MathWorks, Inc., Natick, MA) named OilDropSpec (Sesterhenn, 2012). The program normalized the long wavelength arm of each oil droplet spectra to one, determined the wavelength at which the absorbance = 0.5 (λ_mid_), fitted a trend line to the absorbance data 10 nm on either side of λ_mid_, recorded the intercept, slope, and R^2^ parameters of the trend line, determined the wavelength at which the absorbance = 1 (λ_cut_) using the trend line parameters, calculated λ_0_, *b*, *B*_mid_, and provided these values as an output saved as a Microsoft Excel© file.

We used cluster analysis (*proc fastclus* procedure in SAS 9.2) as a way to statistically verify the identity of the four single peak oil droplet types found in the cowbird retina. We forced four clusters in the analysis, one for each of the four oil droplet types (C, P, Y, and R), excluding the P2 double peaked oil droplet as its identity is visually distinct from the other types. This cluster analysis used four spectral parameters to cluster the oil droplets, λ_cut_, λ_mid_, intercept, and slope, which were determined for each oil droplet through OilDropSpec (Sesterhenn, 2012). Each oil droplet was assigned into one of the four clusters using the nearest centroid sorting based on the four spectral parameters, resulting in the final characterization of the oil droplet spectra. This was primarily used to determine the identity of a few C/P-type oil droplet spectra that could not be identified by their λ_cut_ values alone. After this analysis, we presented the absorbance of each visual pigment and oil droplet type by averaging individual raw absorbance spectra and then normalizing the resulting average spectrum to one (Beason and Loew, 2008).

**Results**

We collected visual pigment absorbance data from 18 cones (Table 1) and 17 rods, corresponding to five cowbirds (4 females, 1 male, and 1 individual of unknown sex). Template fitting revealed that all the single cone visual pigments were A1-rhodopsin in shape. Brown-headed cowbirds have four separate classes of single-cone photoreceptors (Figure 1a-d; Hart 2001a) and one rod (Figure 1e). The average peak absorbance (λ_max_) values for each single cone visual pigments were: 369 nm (UVS), 475 nm (SWS), 506 nm (MWS), and 573 nm (LWS) (Figure 1a-d, f). We were not able to measure the double cone visual pigments during the microspectrophotometric analysis. The average peak absorbance (λ_max_) for the RH1 (rod) visual pigment was 507 nm (Figure 1e-f).

We collected oil droplet absorbance data from 101 oil droplets from five birds (4 females and 1 male) containing 5 distinct types: T-type, C-type, P-type (two variants labeled P1 [one absorbance peak] and P2 [two absorbance peaks]), Y-type, and R-type (Figure 2a-b). We classified these spectra initially based on their shape and λ_cut_ values (Bowmaker et al. 1997; Hart, 2001a), but confirmed their identity using cluster analysis (except the P2 variant; Figure 2d). The ratio of between-cluster variance to within-cluster variance (rsq ratio; *R^2^*/(1 – *R^2^*)) revealed that λ_cut_ had the highest contribution to clustering (rsq ratio = 51.66) followed by λ_mid_ (rsq ratio = 39.53), slope (rsq ratio = 0.22), and the intercept (rsq ratio = 0.09). Using λ_cut_ and λ_mid_ as the distinguishing spectral parameters, we were able to achieve 92.4% agreement in oil droplet identification when performed qualitatively and when performed using cluster analysis, with the remaining 7.6% accounting for the C/P oil droplet spectra which were not able to be identified with confidence qualitatively. The P2 variant was easily identified by the double peaked oil droplet. We distinguished the two peaks as the higher absorbance/lower wavelength peak (P2a) and the lower absorbance/higher wavelength peak (P2b) (Figure 2a). We maintained the distinction between the P1 and P2 variants to show the variation of the principal oil droplets, as they are highly variable in their spectral shape and location in the retina (Hart, 2001b).

The λ_cut_ parameter of four oil droplet types was determined as 418 nm (C-type); 436 nm (P1-type); 448 nm (P2a-type) and 487 nm (P2b-type); 516 nm (Y-type); and 576 nm (R-Type) (Table 1, Figure 2a-b). We could not determine the λ parameters in the T-type oil droplet because they do not absorb light above 350nm due to the lack of carotenoids in the oil droplet (Hart, 2001a). When the absorbance of the oil droplets and ocular media is taken into account along with the sensitivity of the visual pigments, the peak sensitivity of each photoreceptor type (λ_max_) becomes: 373 nm (UVS), 477 nm (SWS), 545 nm (MWS), 609 nm (LWS), and 574 nm for the double cone (Figure 2c).

**References**

Beason, R.C. & Loew, E.R. 2008. Visual pigment and oil droplet characteristics of the bobolink (*Dolichonyx oryzivorus*), a new world migratory bird. Vision Research 48: 1-8.

Bowmaker, J.K, Heath, L.A., Wilkie, S.E. & Hunt, D.M. 1997. Visual pigments and oil droplets from six classes of photoreceptor in the retinas of birds. Vision Research 37(16): 2183-2194.

Crescitelli, F. 1972. The visual cells and visual pigments of the vertebrate eye. Handbook of Sensory Physiology 2 (1): 245-363.

Cuthill, I.C. 2006. Color perception. In: GE Hill & KJ McGraw (eds). Bird coloration: mechanisms and measurements. Vol 1. Harvard University Press, Cambridge. 3-40 p.

Govardovskii, V.I., Fyhrquist, N., Reuter, T., Kuzmin, D.G. & Donner, K. 2000. In search of the visual pigment. Visual Neuroscience 17: 509-528.

Hart, N.S. 2001a. The visual ecology of avian photoreceptors. Progress in Retinal and Eye Research 1(5): 675-703.

Hart, N.S. 2001b. Variations in cone photoreceptor abundance and the visual ecology of birds. Journal of Comparative Physiology A 187: 685-698.

Hart, N.S. & Vorobyev, M. 2005. Modelling oil droplet absorption spectra and spectral sensitivities of bird cone photoreceptors. Journal of Comparative Physiology A 191: 381-392.

Liebman, P.A. 1972. Microspectrophotometry of Photoreceptors. Handbook of Sensory Physiology 2 (1): 481-528.

Lipetz, L.E. 1984. A new method for determining peak absorbance of dense pigment samples and its application to the cone oil droplets of *Emydoidea blandingii*. Vision Research 24: 597–604.

Loew, E.R. 1988-1992. MSP Control and Analysis Program [computer software]. Ithaca: New York State College of Veterinary Medicine.

McFarland, W.N. & Loew, E.R. 1994. Ultraviolet visual pigments in marine fishes of the family Pomacentridae. Vision Research 34: 1393-1396.

Sesterhenn, T. 2012. OilDropSpec (version 4.0) [computer software]. Available from: http://estebanfj.bio.purdue.edu/oildropspec.html

Ullmann, J.F.P., Moore, B.A., Temple, S.E., Fernández-Juricic, E. & Collin, S.P. 2012. The retinal wholemount technique: a window to understanding the brain and behaviour. Brain, Behavior & Evolution 79: 26-44.

Table 1. Mean ± standard error of λ_max_ values of the rod and cone visual pigments in the Brown-headed cowbird. Mean ± standard error of λ_mid_, λ_cut_, λ_0_, *b*, *B*_mid_ values of the five individual oil droplet types associated with the visual pigments as well as the average oil droplet spectrum for each oil droplet type.

| \|  \| Rod  RH1 \| UVS \| Single  SWS \| Cone  MWS \| LWS \|  \| Double Cone \|  \| \| --- \| --- \| --- \| --- \| --- \| --- \| --- \| --- \| --- \| \| Visual Pigments \|  \|  \|  \|  \|  \|  \|  \|  \| \| Mean λ_max_ of spectra (nm) \| 507± 0.9 \| 369 ± 2.2 \| 475 \| 506 ± 2.0 \| 573 ± 4.7 \|  \|  \|  \| \| Number of outer segments \| 17 \| 5 \| 1 \| 8 \| 4 \|  \|  \|  \| \|  \|  \|  \|  \|  \|  \|  \|  \|  \| \|  \|  \|  \|  \|  \|  \|  \|  \|  \| \|  \|  \| **T-Type** \| **C-Type** \| **Y-Type** \| **R-Type** \| **P1-Type** \| **P2-Type** \| \| \|  \|  \|  \|  \|  \|  \|  \| **a** \| **b** \| \|  \|  \|  \|  \|  \|  \|  \|  \|  \| \| Oil Droplets \|  \|  \|  \|  \|  \|  \|  \|  \| \| Mean λ_mid_ (nm) \|  \|  \| 434 ± 1.0 \| 536 ± 1.9 \| 595 ± 1.8 \| 454 ± 1.8 \| 460 ± 1.5 \| 502 ± 2.4 \| \| Mean λ_cut_ (nm) \|  \|  \| 418 ± 0.7 \| 516 ± 1.7 \| 576 ± 1.5 \| 436 ± 1.5 \| 448 ± 1.5 \| 487 ± 1.9 \| \| Mean λ_0_ (nm) \|  \|  \| 430 ± 0.9 \| 531 ± 1.8 \| 590 ± 1.7 \| 449 ± 1.7 \| 457 ± 1.5 \| 498 ± 1.8 \| \| Mean b \|  \|  \| 0.099 ± 0.005 \| 0.074 ± 0.002 \| 0.095 ± 0.002 \| 0.078 ± 0.002 \| 0.129 ± 0.011 \| 0.098 ± 0.008 \| \| Mean B_mid_ \|  \|  \| 0.034 ± 0.002 \| 0.026 ± 0.001 \| 0.033 ± 0.001 \| 0.027 ± 0.001 \| 0.045 ± 0.004 \| 0.034 ± 0.003 \| \| λ_mid_ of mean absorbance spectrum (nm) \|  \|  \| 433 \| 530 \| 590 \| 448 \| 456 \| 498 \| \| λ_cut_ of mean absorbance spectrum (nm) \|  \|  \| 416 \| 513 \| 574 \| 432 \| 447 \| 488 \| \| λ_0_ of mean absorbance spectrum (nm) \|  \|  \| 429 \| 526 \| 586 \| 444 \| 454 \| 495 \| \| b of mean absorbance spectrum \|  \|  \| 0.087 \| 0.084 \| 0.092 \| 0.089 \| 0.161 \| 0.138 \| \| B_mid_ of mean absorbance spectrum \|  \|  \| 0.030 \| 0.029 \| 0.032 \| 0.031 \| 0.056 \| 0.048 \| \| Number of Oil Droplets \|  \| 4 \| 30 \| 32 \| 14 \| 19 \| 6 \|  \| \|  \|  \|  \|  \|  \|  \|  \|  \|  \| |
| --- | --- | --- | --- | --- | --- | --- | --- | --- | --- | --- | --- | --- | --- | --- | --- | --- | --- | --- | --- | --- | --- | --- | --- | --- | --- | --- | --- | --- | --- | --- | --- | --- | --- | --- | --- | --- | --- | --- | --- | --- | --- | --- | --- | --- | --- | --- | --- | --- | --- | --- | --- | --- | --- | --- | --- | --- | --- | --- | --- | --- | --- | --- | --- | --- | --- | --- | --- | --- | --- | --- | --- | --- | --- | --- | --- | --- | --- | --- | --- | --- | --- | --- | --- | --- | --- | --- | --- | --- | --- | --- | --- | --- | --- | --- | --- | --- | --- | --- | --- | --- | --- | --- | --- | --- | --- | --- | --- | --- | --- | --- | --- | --- | --- | --- | --- | --- | --- | --- | --- | --- | --- | --- | --- | --- | --- | --- | --- | --- | --- | --- | --- | --- | --- | --- | --- | --- | --- | --- | --- | --- | --- | --- | --- | --- | --- | --- | --- | --- | --- | --- | --- | --- | --- | --- | --- | --- | --- | --- | --- | --- | --- | --- | --- | --- | --- | --- | --- | --- | --- | --- | --- | --- | --- | --- | --- | --- | --- | --- | --- | --- | --- | --- | --- | --- | --- | --- | --- | --- | --- | --- | --- | --- | --- | --- | --- | --- | --- | --- |

Figure legends

Fig. 1. Normalized absorbance spectra of the brown-headed cowbird photoreceptors: a) ultraviolet sensitive cone (UVS), b) short-wavelength sensitive cone (SWS), c) medium-wavelength sensitive cone (MWS), d) long-wavelength sensitive cone (LWS), and e) rod (RH1). In (a-e), black lines are the visual pigment λ_max_ specific A1-rhodopsin pigment template created using Govardovskii et al. 2000. In f), the frequency diagram of λ_max_ values for each type of visual pigment (UVS, SWS, MWS, RH1, and LWS respectively) that were used to calculate the mean λ_max_ value of each visual pigment type.

Fig. 2. Brown-headed cowbird oil droplets. In a), the normalized absorbance spectra of the, C-Type (blue), P1-type (orange), P2-type (orange-brown), Y-type (yellow), R-type (red), and T-type (purple). The T-type spectrum shows the raw absolute absorbance of this type for ease of viewing. In b), the frequency diagram of λ_cut_ values for each type of oil droplet (C-, P1-, P2a-, P2b-, Y-, and R-type respectively) that were used to calculate the mean λ_cut_ value of each oil droplet type. In c), the spectral tuning effects of the oil droplets on the four classes of single cone (Figure 1a-e) and one double cone (Table 1) visual pigment found in the Brown-headed cowbird. The dotted line spectra represent the visual pigment curves of each photoreceptor. The solid line spectra represent the effect of the oil droplets and ocular media transmittance on the sensitivity of the entire photoreceptor on the cowbird retina. Visual pigment curves were created using A1-rhodopsin pigment templates from Govardovskii et al. 2000 and oil droplet transmittance templates from Hart and Vorobyev (2005). Double cone spectral sensitivity is based solely on the P1 type oil droplet. The color of the lines corresponds to specific photoreceptor types; UVS (violet), SWS (blue), MWS (green), LWS (red), double cone (LWS [red] pigment with P1-type [orange] oil droplet). In d), the resulting clusters from oil droplet cluster analysis based on λ_mid_ and λ_cut_ values (C-Type is blue stars, P1-Type is orange squares, Y-Type is yellow diamonds, and R-Type is red triangles).

Fig. 1

Fig. 2
