# Supplementary material for: Do Male and Female Cowbirds See Their World Differently? Implications for Sex Differences in the Sensory System of an Avian Brood Parasite
Source: PLoS One. 2013 Mar 27;8(3):e58985. doi: 10.1371/journal.pone.0058985 (PMC3609808; doi:10.1371/journal.pone.0058985)
Supplement: Appendix S3 — Perceptual modeling of the visual stimuli. (DOCX) [file pone.0058985.s003.docx]

**Appendix S3: Perceptual modeling of the visual stimuli**

We estimated chromatic contrasts for each of the 20 brown-headed cowbirds (10 males and 10 females) that were used to estimate the density of cone photoreceptors. Having individual estimates of chromatic contrast allowed us to test statistically for sex differences (see main text). We estimated chromatic contrast separately for the central and peripheral retina and for the two objects presented to the birds during the behavioral experiment (colored and black cubes).

To estimate chromatic contrast we used Vorobyev and Osorio (2008)’s physiological model for tetrachromats using Avicol v6 (Gomez, 2006). We included in the model the following parameters: (1) the wavelength-specific capture probabilities (*S_i_(λ)*) based on the averaged sensitivity of visual pigments (λ_max_) and oil droplets (λ_cut_) from our brown-headed cowbird microspectrophotometric data (Appendix S2), (2) relative cone photoreceptor densities (for estimation of neural noise or *e_i_; see below*) of the individual cowbirds, (3) irradiance of ambient light (*I(λ)*), and (4) reflectance of the cubes and visual background (*R(λ)*).

To calculate the wavelength-specific capture probabilities ((*S_i_(λ)*); C_r_(*λ*) in Endler and Mielke 2005) for each single cone photoreceptor type (UVS, SWS, MWS, and LWS), as well as the double cone type, we used: (1) the absorbance templates developed from the average sensitivities of the visual pigments (λ_max_) from our cowbird data (Appendix S2), (2) the transmittance templates for each type of oil droplet (based on equation [17] in Hart and Vorobyev 2005) using λ_cut_ and *B_mid_* from our cowbird data (Appendix S2), and (3) the transmittance of the ocular media using equation [7] from Endler & Mielke (2005). We then normalized the Cr(*λ*) spectra to an area of one to obtain the data we input into Avicol v6. For the double cones, we used only the spectra from the P1 oil droplet type, and we assumed that they contained the LWS visual pigment (following Hart 2001). We did not use the double-peaked P2-type of oil droplet because Avicol v6 limits the number of double-cone spectra that can be used.

Cone photoreceptor densities, used in estimating neural noise (*e_i_*) in the contrast models, were calculated based on oil droplet densities at the center and periphery of the retina of each individual (see main text). We modeled the noise of each photoreceptor type as neural noise only, independent of light intensity, using the SWS, MWS and LWS cone densities (relative to UVS cone densities), and a Weber fraction of 0.05 (typical of a UVS system, Håstad et al. 2005).

We measured irradiance (*I(λ)*) and reflectance (*R(λ)*) with a StellarNet EPP2000 portable spectroradiometer (StellarNet-Inc., Tampa, FL). For irradiance, we positioned the probe on the bottom of the enclosure in the position of the cube (see main text). We took 10 measures of irradiance: with the probe at the cardinal points (North, South, East, West), each at 45° and 90° from the light source, plus two measures with the probe directly aimed at the light source. Irradiance was measured in increments of 0.5 nm, from 300-700 nm, using a cosine corrected sensor calibrated with a standardized light source in Watts m^-2^. Measurements from all 10 positions were then averaged together, and converted to µMol m^-2^s^-1^nm^-1^ for use as input for Avicol v6.

We measured reflectance of the cubes and the paper background at the bottom of the enclosure using the same spectroradiometer in increments of 0.5 nm, from 300-700 nm, using a Tungsten Krypton light and Deuterium light source simultaneously and a micron fiber optic probe. Calibrations were done against a standard white surface (97% reflectance of white light), with the light source on for the light standard, and the light source off for the dark standard. The probe was held at a 45° against the surface of the cube or the paper background. Sixty reflectance measurements were taken on the black cube (10 per side) and 20 on the paper background. Each side of the colored cube had three blue squares, three green squares, two red squares, and one yellow square (Fig. 3.1). The yellow square was in the center, not sharing any edges with the brown background; therefore we did not include the yellow reflectance in the contrast calculations. Two reflectance measurements were taken per square, per side, totaling 36 measurements of blue and green squares each and 24 measurements of the red squares. For each color, we averaged the wavelength-specific measurements, and used these averaged spectra in calculating pair-wise contrasts of each color with the visual background using Avicol v6.

We used the program Avicol v6 (Gomez, 2006) to estimate chromatic contrast. In Avicol, we chose Vorobyev and Osorio’s physiological model for tetrachromats, which estimates the distance between the quantum catch of the stimuli/object and the background in a tetrahedral receptor colorspace (Vorobyev & Osorio 1998).

The model states that the color stimulus of object *x* is defined by the quantum catch of each photoreceptor class *i*:

$Q_{i,x}= \int_{{}_{min}}^{{}_{max}} R_{x}\left( \right)S_{i}()I\left( \right)d()$ Eq. 1

where *λ* is wavelength, $R_{x}\left( \right)$ is the wavelength-specific reflectance spectrum of the object $x$, $S_{i}()$ is the wavelength-specific spectral sensitivity of receptor $i$, and $I\left( \right)$ is the wavelength-specific spectrum of ambient light. Integration is over the visible spectrum of the organism, and in the case of birds this would be from 300 to 700 nm.

The relationship between the quantum catch of two stimuli (*a* and *b*) for photoreceptor class *i* is:

$f_{i}=\ln\left( Q_{i,a} \right)-\ln\left( Q_{i,b} \right)=ln\left( \frac{Q_{i,a}}{Q_{i,b}} \right)$ Eq. 2

Each single photoreceptor has associated “noise” in the information it conveys. The standard deviation of the noise of a single photoreceptor cell is represented by $v_{i}$. The effect of this noise on color perception is decreased by the presence of many photoreceptors of a given class, such that the overall noise for a given class of photoreceptors is:

$e_{i}= v_{i}/\sqrt{{}_{i}}$ Eq. 3

where ${}_{i}$ is the density of photoreceptors of type $i$ relative to the UVS densities, and $e_{i}$ is the noise of type $i$ of the four photoreceptor classes. The term $v_{i}$ is the Weber fraction for the UVS cone, which is the standard deviation of the noise in a single UVS photoreceptor. All relative photoreceptor densities are calculated in relation to the UVS cone density, such that ${}_{UVS}$=1,${}_{SWS}$=the density of SWS cones divided by the density of UVS cones, and so on. Then, $e_{UVS}$ is equal to the Weber fraction, but $e_{i}$ for the other cones may vary according to${}_{i}$.

Incorporating the noise and the difference in quantum catch between two stimuli, the square of chromatic contrast ($S$) of two stimuli in a tetrachromatic system is given by:

${(S)}^{2}=\left( {(e_{1}e_{2})}^{2}{(f_{4}-f_{3})}^{2}+{(e_{1}e_{3})}^{2}{(f_{4}-f_{2})}^{2}+{(e_{1}e_{4})}^{2}{(f_{3}-f_{2})}^{2}+{(e_{2}e_{3})}^{2}{(f_{4}-f_{1})}^{2}+{(e_{2}e_{4})}^{2}{(f_{3}-f_{1})}^{2}+{(e_{3}e_{4})}^{2}{(f_{2}-f_{1})}^{2} \right)/\left( \left( e_{1}e_{2}e_{3} \right)^{2}+\left( e_{1}e_{2}e_{4} \right)^{2}+\left( e_{1}e_{3}e_{4} \right)^{2}+\left( e_{2}e_{3}e_{4} \right)^{2} \right)$ Eq. 4

Chromatic contrast is in units of just noticeable difference (or JND), representing chromatic discriminability. This is signal-to-noise ratio, where the signal is the difference between the object reflectance and the background reflectance, and the noise is that produced by the photoreceptors. In the model presented by Vorobyev et al. (1998), the noise in the photoreceptors is set by: (1) the standard deviation of the noise assumed in a single photoreceptor ($v_{i}$), (2) the spectral sensitivities of the photoreceptors ($S_{i}()$), and (3) the relative densities of the photoreceptors (${}_{i}$). Then, there is a certain distance between the two hues being compared (the cube and the background), based on the reflectance spectra and the ambient light, where the distance between the two hues is equal to the noise generated in the photoreceptors. For the few estimates that have been obtained on noise in the photoreceptors (Maier 1992), $S$ = 1 JND corresponds to the threshold of discriminability. This JND =1 is used as a threshold convention for most species due to the little data on photoreceptor noise.

Consequently, $S$ < 1 JND represents a situation in which the distance between the two hues is less than the noise in the photoreceptors, and therefore the two hues are assumed not be discriminable. It has been suggested that JNDs between 1 and 4 indicate that the object can be distinguished but with some difficulty (Siddiqi et al. 2004). Finally, JNDs > 4 suggest that animals can easily distinguish the object from the visual background (Siddiqi et al. 2004), and more easily as JNDs increase, since the distance between the spectra of the object and the background becomes increasingly higher than the noise in the photoreceptors (Vorobyev et al. 1998).

We calculated chromatic contrast, ${(S)}^{2}$, for the visual background versus the black cube, and pair-wise between the background versus the colored cube blue squares, red squares and green squares. To obtain a final single chromatic contrast value for the colored cube, we first obtained weighted contrasts for each color-background combination. These weighted contrasts were calculated by multiplying the pair-wise contrasts of the colors versus the background (produced by Avicol v6 and explained above) by the relative contributions (blue: 0.42, green: 0.333, and red: 0.25) of each color to the total contrast of the colored cube against the background (Fig. 3.1). These relative contributions were based on the number of edges that each color shared with the brown paper background when viewing one side of the cube (Fig. 3.1): blue (n=5), green (n=4), red (n=3). We then summed the weighted contrasts in order to obtain a single measure of chromatic contrast for the colored cube.

One of the shortcomings of this analysis is that fact that the estimates of photoreceptor density corresponded to individuals different from the ones used in the behavioral experiment. However, our intention was to focus on sex differences, which were found in both the physiology and behavioral studies (see also Discussion).

**Literature cited**

Endler, J.A. & Mielke, Jr., P.W. 2005. Comparing entire colour patterns as birds see them. Biological Journal of the Linnean Society 86: 405-431.

Gomez, D. 2006. AVICOL, a program to analyse spectrometric data. Last update October 2011. Free executable available at http://sites.google.com/site/avicolprogram/ or from the author at dodogomez@yahoo.fr

Håstad, O., Victorsson, J. & Ödeen, A. 2005. Differences in color vision make passerines less conspicuous in the eyes of their predators. Proceedings of the National Academy of Sciences 102: 6391-6394.

Hart, N.S. 2001. The visual ecology of avian photoreceptors. Progress in Retinal and Eye Research 20: 675-703.

Hart, N.S. & Vorobyev, M. 2005. Modelling oil droplet absorption spectral sensitivities of bird cone photoreceptors. Journal of Comparative Physiology 191: 381-392.

Maier, E. J. 1992. Spectral sensitivites including the ultraviolet of the passeriform bird *Leiothrix lutea*. Journal of Comparative Physiology A 170: 709-714.

Moore, B.A., Baumhardt, P., Doppler, M., Randolet, J., Blackwell, B.F., DeVault, T.L., Loew, E.R. & E. Fernández-Juricic. 2012. Oblique color vision in an open-habitat bird: spectral sensitivity, photoreceptor distribution, and behavioral implications. Journal of Experimental Biology 215: 3442-3452.

Siddiqi, A., Cronin, T.W., Loew, E.R., Vorobyev, M. & Summers, K. 2004. Interspecific and intraspecific views of color signals in the strawberry poison frog *Dendrobates pumilio*. Journal of Experimental Biology 207: 2471-2485.

Vorobyev, M. & Osorio, D. 1998. Receptor noise as a determinant of colour thresholds. Proceedings of the Royal Society of London B 265: 351-358.

Vorobyev, M., Osorio, D., Bennett, A. T. D., Marshall, N. J. & Cuthill, I. C. 1998. Tetrachromacy, oil droplets and bird plumage colours. Journal of Comparative Physiology A 183: 621-633.

Figure legend

Fig. 3.1. Top-view of the colored cube against the paper brown background, showing the pattern of edges shared between colored squares and the background.

Fig. 3.1

|  | brown | brown | brown |  |
| --- | --- | --- | --- | --- |
| Brown | blue | green | blue | brown |
| brown | red | yellow | green | brown |
| brown | green | blue | red | brown |
|  | brown | brown | brown |  |
